# The *in vivo* dsRNA Cleavage Has Sequence Preference in Insects

**DOI:** 10.3389/fphys.2018.01768

**Published:** 2018-12-10

**Authors:** Ruobing Guan, Shaoru Hu, Haichao Li, Zhenying Shi, Xuexia Miao

**Affiliations:** ^1^Key Laboratory of Insect Developmental and Evolutionary Biology, Institute of Plant Physiology and Ecology, Shanghai Institutes for Biological Sciences, Chinese Academy of Sciences, Shanghai, China; ^2^State key Laboratory of Wheat and Maize Crop Science, College of Plant Protection, Henan Agricultural University, Zhengzhou, China; ^3^University of the Chinese Academy of Sciences, Beijing, China

**Keywords:** insect, RNA interference, *in vivo* dsRNA-processing, siRNA, sequence-specific cleavage

## Abstract

Exogenous dsRNA enters the insect body and can induce the RNAi effect only when it is cleaved into siRNA. However, what kinds of base composition are easier to cut and what kinds of siRNA will be produced *in vivo* is largely unknown. In this study, we found that dsRNA processing into siRNA has sequence preference and regularity in insects. We injected 0.04 mg/g dsRNA into Asian corn borers or cotton bollworms according to their body weight, and then the siRNAs produced *in vivo* were analyzed by RNA-Seq. We discovered that a large number of siRNAs were produced with GGU nucleotide residues at the 5′- and 3′-ends and produced a siRNA peak on the sequence. Once the GGU site is mutated, the number of siRNAs will decrease significantly and the siRNA peak will also lost. However, in the red flour beetle, a member of Coleoptera, dsRNA was cut at more diverse sites, such as AAG, GUG, and GUU; more importantly, these enzyme restriction sites have a high conservation base of A/U. Our discovery regarding dsRNA *in vivo* cleavage preference and regularity will help us understand the RNAi mechanism and its application.

## Introduction

RNA interference (RNAi) technology is widely used in scientific research as a genetic tool (Boettcher and McManus, 2015; Blake et al., 2017). It is more likely to be used as a new approach in agricultural pest control (Burand and Hunter, 2013; Kim et al., 2015; Joga et al., 2016). RNAi can be triggered by introducing double-stranded RNA (dsRNA), which is processed into effective small interfering RNAs (siRNAs) by the Dicer enzyme. Then, the generated siRNAs are incorporated into the RISC complex with other proteins, enter into the subsequent RNAi pathway, and then cause the gene silencing effect (Fire et al., 1998; Tijsterman and Plasterk, 2004; Winter et al., 2009). Therefore, the Dicer enzyme processing of the dsRNA into siRNAs is the key step in the RNAi pathway; however, it is not clear how dsRNA is recognized and cleavaged by the Dicer enzyme, or what kinds of siRNAs will be produced *in vivo*.

A previous *in vitro* study indicated that the PAZ domain of Dicer is capable of recognizing the 3′-overhang structure and the 5′-phosphate monoester structures of the dsRNA. Dicer selects cleavage sites by measuring a set distance (∼21 nucleotides) from the 3′- or 5′-end to ensure the precise and effective biogenesis of siRNAs. The PAZ domain is crucial for the siRNA production process. Mutations in the PAZ domain can decrease siRNA length fidelity and RNAi silencing activity *in vivo* (Kandasamy and Fukunaga, 2016). The 3′-counting rule (Zhang et al., 2004; MacRae et al., 2006, 2007) and the 5′-counting rule (Park et al., 2011) have been proposed to explain how Dicer enzymes process dsRNA. In addition to the ends of small hairpin RNAs (shRNAs)/pre-miRNAs, Dicer can recognize the loop/bulge structures for accurate processing. Thus, the loop counting rule has also been proposed (Gu et al., 2012). These results can explain the siRNA’s length and the initiating mode when dsRNA is processed by the Dicer enzyme.

In addition, the studies from Vermeulen et al. (2005) indicated that Dicer has sequence preferences when processing dsRNAs. Therefore, Dicer recognizes the preferred nucleotide residues on the dsRNA and then processes them into siRNA. A recent result has confirmed that Dicer-like enzymes have sequence cleavage preferences in *Paramecium* (Hoehener et al., 2018). Besides this, the Mini-III RNase family protein BsMiniIII in *Bacillus subtilis* is capable of cleaving a long dsRNA substrate in an ACCU/AGGU sequence-specific manner (Glow et al., 2015). Different Dicer-like enzymes or Mini-III RNases have different cleavage capacities on nucleotide bases (Glow et al., 2016; Hoehener et al., 2018). These results illustrated that different RNase III protein families have different preference recognition sites. Therefore, we hypothesized that the dsRNA-processing model was based on the RNase III family proteins and may have some regularity. However, our hypothesis is based on the *in vitro* study results. When a dsRNA segment entered an organism in a complex *in vivo* environment, what kinds of siRNA can be processed for the RNAi pathway? And whether its processing mode has sequence preference is largely unknown.

Here, using a high-throughput small RNA sequencing and bioinformatics analysis strategy, we dissected the *in vivo* dsRNA-processing mode. The Asian corn borer (*Ostrinia furnacalis*) and cotton bollworm (*Helicoverpa armigera*) were selected as models of Lepidoptera insects. We discovered the *in vivo* rule of dsRNA processing in insects. dsRNA processing into small RNA is only related to sequence composition and is not related to the sequence length. GGU was the preferred three-nucleotide digestion site in these two Lepidoptera insects. However, in the red flour beetle (*Tribolium castaneum*), a coleopteran insect, the dsRNA was cut at more diverse sites, such as AAG, GUG, and GUU. These results indicated that the dsRNA processing mode is not only related with the sequence composition, but also related to the *in vivo* environment in different organisms. This is probably a major reason for different RNAi efficiencies in different insect species.

## Materials and Methods

### Insect Culturing

The Asian corn borer (*O. furnacalis)* and cotton bollworm (*H. armigera*) eggs were originally obtained from fields in Shanghai, China and reared in the laboratory at 25 ± 1°C and 75% relative humidity under a 14/10 h light/dark photoperiod. The larvae were fed on a modified artificial diet (Wang et al., 2011).

The red flour beetle was obtained from the laboratory of Dr. Ling’s at the Key Laboratory of Insect Developmental and Evolutionary Biology at the Shanghai Institute of Plant Physiology and Ecology. They were reared on whole wheat flour containing 5% brewer’s yeast at 30°C under a 14/10 h light/dark photoperiod.

### dsRNA Preparation

DsRNAs were synthesized using the MEGAscript RNAi Kit (Ambion, Huntingdon, United Kingdom) according to the manufacturer’s instructions. T7 promoter sequences were tailed to the 5′ ends of the DNA templates by PCR amplifications. Double-stranded enhanced green fluorescent protein (ds*EGFP*) (GenBank accession no. MF169984) was generated using pPigbacA3EGFP as the template. All the primer sequences are listed in Supplementary Table 1. Template DNA and single-stranded RNA were removed from the transcription reaction through DNase and RNase treatments, respectively. dsRNA was purified using MEGAclear columns (Ambion, Austin, TX, United States) and eluted in nuclease free water. dsRNA concentrations were measured using a BioPhotometer (Eppendorf, Hamburg, Germany).

### Microinjection and Sample Collection

The fifth-instar larvae of the Asian corn borer (*O. furnacalis*), the third-instar larvae of the cotton bollworm (*H. armigera*), and the fifth-instar larvae of the red flour beetle (*T. castaneum*) were used as experimental materials. Each gram of insect was injected with 0.04 mg dsRNA in the posterior abdominal segment using a capillary needle. Three *O. furnacalis* or *H. armigera* larvae were treated, twelve *T. castaneum* larvae regarded as one treatment were treated, and each treatment was repeated three times. The untreated fifth-instar *O. furnacalis* larvae were regarded as the control group. Four hours after the injections, samples were collected, frozen in liquid nitrogen, and stored at -80°C until RNA extraction.

### RNA Isolation and Small RNA Sequencing

Total RNAs were isolated using TRIzol reagent (Invitrogen) according to the manufacturer’s instructions. Samples were treated with RNase-free DNaseI (New England BioLabs, Ipswich, MA, United States) for 30 min at 37°C to remove residual DNA prior to small RNA sequencing. Samples were sequenced using an Illumina HiSeq 2000 analyzer at BGI (Shenzhen, China). The sequencing information is listed in Supplementary Table 2.

### Small RNA Sequencing Analysis

In this research, small RNAs from related treatments were re-mapped onto the dsRNA sequence using local BLASTn (*E*-value < 10^-5^); only one base mismatch was allowed during calculation. The type and number of small RNAs that were processed by dsRNA were calculated and the distribution of small RNAs were subsequently analyzed. The 19–25 nt long small RNAs were used for further analysis. The perl SVG module was used to make a graph, with the x-axis representing the dsRNA sequence and the y-axis representing the depth of sequencing (amount of mapped small RNA).

When a small RNA was mapped on the reference dsRNA sequence, the dsRNA cleavage sites were determined. Three nucleotide bases at the front and back of the 5′-end of a small RNA were named as 5′ cleavage site, three nucleotide bases at the front and back of the 3′-end of a small RNA were named as 3′ cleavage site. And then the nucleotide residues of 5′- and 3′-ends cleavage sites were calculated and analyzed.

### Real-Time Quantitative PCR (qRT-PCR)

Total RNA was extracted from pools of three surviving dsRNA-treated larvae using TRIzol reagent (Invitrogen), according to the manufacturer’s instructions. First-strand cDNA was made from 1 μg of RNA primed by oligo (dT)_18_ using M-MLV reverse transcriptase (Takara, Kyoto, Japan). A qRT-PCR assay that amplified multiple genes was performed using SYBR Premix Ex Taq^TM^ II (Takara). To ensure the qRT-PCR’s quality, two or three primer pairs were designed for all the amplification segments, but only one pair was used in the final test. All the primer sequences are listed in Supplementary Table 1. Melting-curve analyses were performed for all the primers. To normalize Ct values obtained for each gene, 18S rRNA expression levels were used (Chapman and Waldenstrom, 2015). The qRT-PCR was carried out using a Mastercycler ep realplex (Eppendorf). All the qPCR assays were repeated three times. The qRT-PCR reactions and data were analyzed according to the methods of Livak and Schmittgen (2001) and Bustin et al. (2009). The qRT-PCR data were analyzed using a one-way analysis of variance (ANOVA) followed by Duncan’s multiple range test to look for treatment effects compared with the untreated control.

## Results

### Target dsRNA Sequence Composition Determines the Amount of Small RNA

**FIGURE 1:**
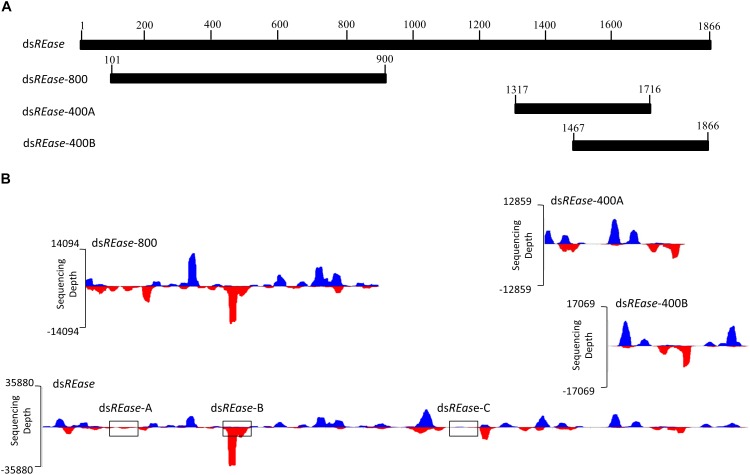
The dsRNA-processing model is closely related to its nucleotide sequence. **(A)** *REase* gene expression levels under different treatments. Fifth instar Asian corn borer larvae were independently injected with 10 μg of ds*REase*, ds*REase*-800, ds*REase*-400A, and ds*REase*-400B. Four hours later, samples were collected and *REase* gene expression levels were determined by qRT-PCR. Compared to the ds*EGFP* treatment, the *REase* gene can be repressed by the four kinds of ds*REase*. **(B)** Processing mode of the four kinds of ds*REase* *in vivo*. Fifth instar Asian corn borer larvae were independently injected with 10 μg of ds*REase*, ds*REase*-800, ds*REase*-400A, and ds*REase*-400B. Four hours later, RNAs were isolated for small RNA sequencing. Small RNAs of 19–25 nt long were re-mapped on the reference sequences (*x*-axis) to produce the graph. Blue peaks indicate that the small RNAs matched on the sense chain. Red peaks indicate that the small RNAs matched on the anti-sense chain. The *x*-axis represents the *REase* sequence, and the y-axis represents the depth of sequencing (amount of mapped small RNA). The three black boxes of ds*REase*-A, ds*REase*-B, and ds*REase*-C were 100 bp sequences in different position for small RNAs analysis. For the statistical results, please see Supplementary Figure 3.

When a dsRNA was introduced into one insect body, what kinds of siRNAs were produced and induced RNA? To analyze this issue, an RNAi efficiency-related nuclease (*REase*) (the full length CDS is 1866 bp) (GenBank accession no. F682492) was selected as a template to synthesize four kinds of dsRNAs. They are ds*REase* (1–1866 bp), ds*REase*-800 (101–900 bp), ds*REase*-400A (1317–1716 bp), and ds*REase*-400B (1467–1866 bp) according to different segments (Figures 1A,B). Then, each kind of dsRNA was injected into a fifth instar Asian corn borer larva (triplicated). After 4 h, total RNA was extracted for qRT-PCR and small RNA sequencing. The qRT-PCR results indicated that all four kinds of ds*REase* suppressed *REase* gene expression levels compared with the ds*EGFP* treatment (Supplementary Figure 1). These results also confirmed that all these four kinds of dsRNA segments can be processed into siRNA *in vivo* and induce target gene RNAi in insect.

To analyze the siRNA produced by these four kinds of dsRNA *in vivo*, small RNAs were sequenced using an Illumina HiSeq 2000 analyzer at BGI (Shenzhen, China). Approximately 90% of the small RNA sequences were 18–30 nt, and 70% were 19–25 nt (Supplementary Figure 2 and Supplementary Table 2). The lengths of these small RNAs conformed to those of siRNAs. Thus, we assumed that most of these small RNAs are siRNAs, which can combine with Argonaute protein, and result in the RNAi effect on the target gene. In this study, 19–25 nt small RNAs were selected and re-mapped on the corresponding dsRNA sequences of ds*REase*, ds*REase*-800, ds*REase*-400A, and ds*REase*-400B (Supplementary Table 3). With the *x*-axis representing the dsRNA sequence, and the y-axis representing the depth of sequencing (amount of mapped small RNA) to make a graphing, a very interesting phenomena was revealed (Figure 1B). All the same dsRNA sequences were processed into similar small RNA *in vivo* by a consistent processing mode. More important is that the small RNA peak always appeared at the same sequence position among the different treatments (Figure 1B). This phenomenon also implied that some dsRNA segments with small RNA peaks will produce large amounts of small RNAs, while other segments without small RNA peaks will only produce a limited amount of small RNAs (Figure 1B), the three black boxes of ds*REase*-A, ds*REase*-B, and ds*REase*-C; for statistical results please see Supplementary Figure 3. At the same time, the control treatment, which was not injected with exogenous dsRNA, only produced a small number of small RNAs that mapped on corresponding dsRNA sequences (Supplementary Table 3). Thus, we confirmed that the mapped small RNAs of 19–25 nt resulted from the dsRNA were injected into the insects’ bodies. Accordingly, we hypothesized that specific nucleotide hot points for digestion may exist when dsRNA is processed into small RNAs *in vivo*.

**FIGURE 2:**
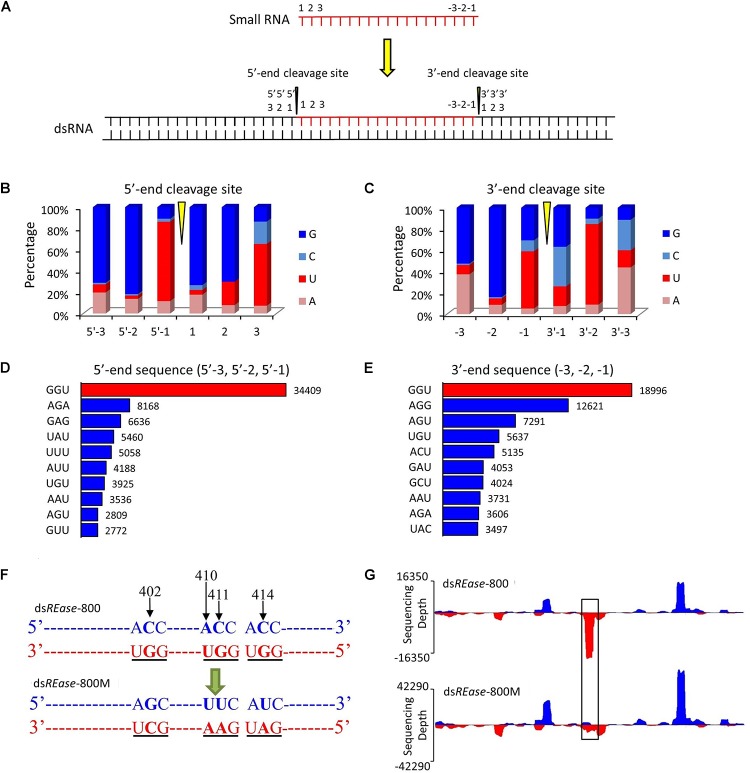
GGU is a major cleavage site for *in vivo* dsRNA-processing into small RNAs. **(A)** Outline of the dsRNA cleavage site analysis. Three nucleotide bases on the 5′- and 3′-ends of a small RNA were labeled as 1, 2, and 3, and -3, -2, and -1, respectively. When one small RNA was mapped on the reference dsRNA sequence, the small RNA cleavage site was revealed. Three nucleotide bases before the 5′-end’s cleavage site were labeled as 5′-3, 5′-2, and 5′-1. Similarly, three nucleotide bases after the small RNA’s 3′-end cleavage site were named as 3′-1, 3′-2, and 3′-3. **(B,C)** Nucleotide components of 5′- and 3′-end cleavage sites of the top 0.1% of small RNAs that match on the reference sequence. **(D,E)** Three nucleotides components of the top ten 5′- and 3′- end cleavage sites of the top 1.0% of 19–25 nt small RNAs. The nucleotide components of the 5′-end sequence represent the information on sites of 5′-3, 5′-2, and 5′-1, and those of 3′-end sequence represent the information on sites of -3, -2, and -1. **(F)** GGU site’s mutant design. One small RNA peak representing an area with three GGU cleavage sites was selected in ds*REase*-800. The site-specific mutations were produced by PCR. The nucleotide site 402, 410, 411, and 414 on the sense chain were mutant from CACC to GUUU. Thus, the three GGU sites on the antisense chain were changed to GCU, GAA, and GAU. **(G)** The small RNA peak was lost when the GGU site was mutant. Four hours later injection with 10 μg ds*REase*-800 with three GGU sites or 10 μg ds*REase*-800M with GGU mutation, total RNA was extracted from the fifth instar Asian corn borer larvae. Then, small RNAs were isolated and sequenced (sequence data are listed in Supplementary Tables 2–5). The 19–25 nt long small RNAs that matched on the reference sequences were used for graphing.

**FIGURE 3:**
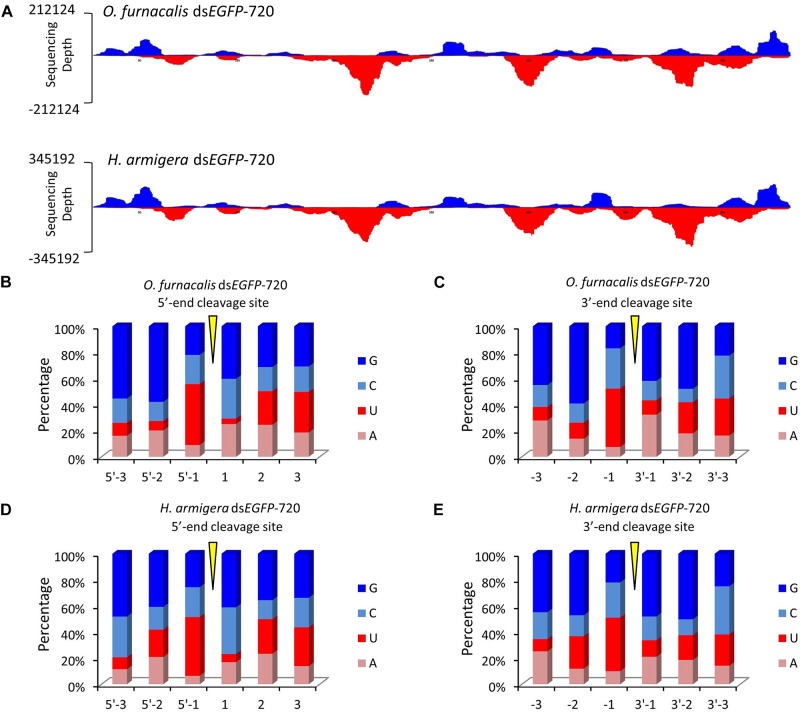
The GGU cleavage site is a universal phenomenon in Lepidoptera. **(A)** Ds*EGFP*-720 revealed similar cleavage modes in the Asian corn borer (*Ostrinia furnacali*s) and in cotton bollworm (*Helicoverpa armigera*) when small RNAs were re-mapped onto the *EGFP* sequence. The *x*-axis represents the *EGFP* sequence, and the *y*-axis represents the depth of sequencing (amount of mapped small RNA). **(B,C)** Nucleotide components of the 5′- and 3′-end cleavage sites of the top 1.0% of small RNAs in the Asian corn borer. **(D,E)** Nucleotide components of the 5′- and 3′-end cleavage sites of top 1.0% of small RNAs in the cotton bollworm.

### GGU Is an Enzyme Digestion Hot Point When dsRNA Is Processed Into Small RNAs

To discover the cleavage hot points when ds*REase* was processed into small RNAs, the types and copy numbers of each kind of small RNA that was mapped on the full- length *REase* gene were thoroughly analyzed. The amount of the total mapped small RNAs was 418,100 (Supplementary Table 3), which belonged to 39,193 different types of small RNAs (Supplementary Table 5). The copy number, amount and percentage of each kind of small RNA are listed in Table 1. More than 82% of small RNAs had copy numbers of less than 10, but they accounted for only ∼18% of the total amount of small RNAs. Only 1.66% of small RNAs had copy numbers of more than 100; however, they accounted for more than 36% of the small RNA amount. These results indicated that a large amount of small RNAs came from the same dsRNA fragment and produced a small RNA peak (Figure 1B and Table 1). Thus, the dsRNA fragments corresponding to the small RNA peak probably exist at a hot point for enzyme cleavage.

**Table 1:** Distributions of 18–42 nt small RNA copy numbers, numbers and percentages after the ds*REase* treatment.

Copy number	Number of each small RNA type	Percent according to copy number	Small RNA number of these kinds RNAs	Percent according to small RNA number
1 10	32360	82.56	76722	18.35
11 100	6168	15.78	187219	44.78
101 500	610	1.56	109255	26.13
501~1000	29	0.07	18661	4.46
> = 1001	8	0.03	26243	6.28
Total	39193	100	418100	100


To discover the hot point where the dsRNA is cut to produce siRNA, a total of 38,263 19–25 nt small RNAs that had more than 500 copy numbers were selected. They account for just 0.10% of the 19–25 nt small RNA type, but their amount represented 10.74% of the total mapped 19–25 nt small RNAs. Subsequently, through a series of analyses, three nucleotide residues at the front and back of 5′- and 3′-end were analyzed in all 38,263 19–25 nt small RNAs (Figure 2A). The statistical analysis suggested that the nucleotide composition of 35,974 (∼94%) small RNAs had cleavage sites of GGU before the 5′-end and on the 3′-end (Figures 2B,C). To further confirm this result, the 1.0% small RNA type (representing 30% of the total amount of 19–25 nt small RNAs) was analyzed, and 64% of the small RNAs had a GGU site before the 5′-end and on the 3′-end (Supplementary Figures 4A,B). The top 10 three-nucleotide combinations in these positions are shown in Figures 2D,E. The GGU sites represent 45 and 28% of all three-nucleotide combinations before the 5′-end and on the 3′-end, respectively.

**FIGURE 4:**
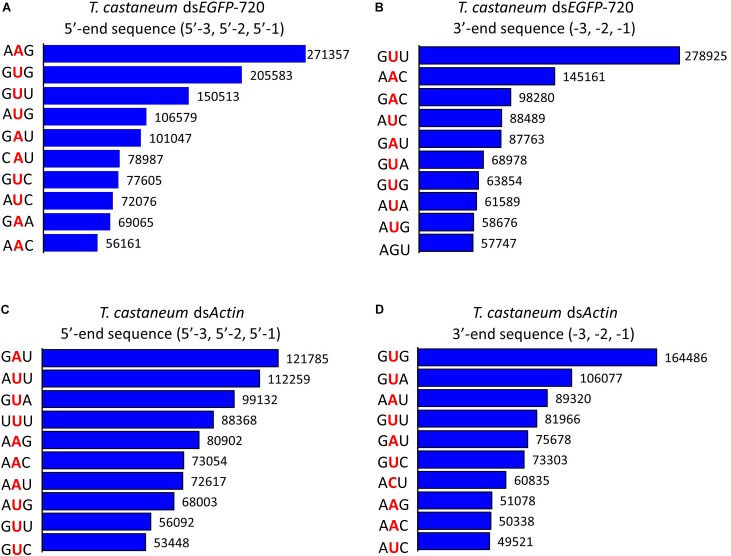
Top 10 three-nucleotide components at the 5′- and 3′-end cleavage sites of the 1.0% of small RNAs in the red flour beetle (*Tribolium castaneum*). **(A,B)** 5′- and 3′-end cleavage sites for ds*EGFP*-720. **(C,D)** 5′- and 3′-end cleavage sites for ds*Actin*.

We also discovered that most of the GGU sites will produce a small RNA peak (Supplementary Figure 5). For confirmation, three GGU sites on the antisense chain of ds*REase*-800 were selected as single base point mutations and the three GGU sites were changed to GCU, GAA, and GAU in ds*REase*-800M (Figure 2F, with black underline). Then, ds*REase*-800 and ds*REase*-800M were each separately injected into individual fifth instar Asian corn borer larvae (Figure 1B). The small RNA peak was lost when GGU sites were changed into other nucleotides (Figure 2G, black box); more importantly, the cleavage mode did not change in other sites on this mutant dsRNA segment. This result further suggested that GGU is an important requirement when dsRNA is being processed into small RNAs *in vivo.*

**FIGURE 5:**
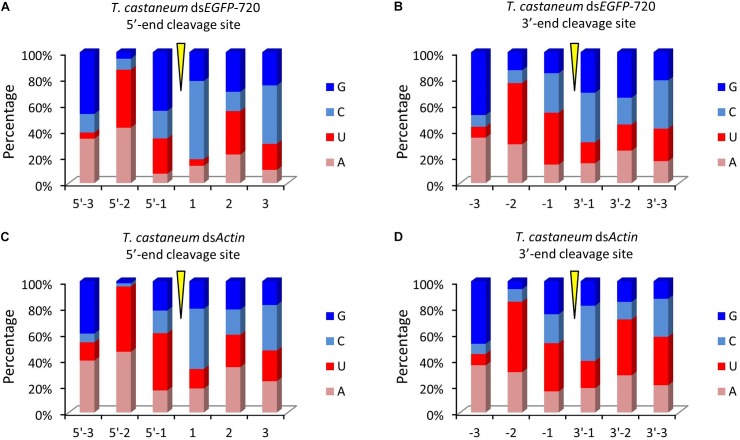
Diversity of small RNA cleavage sites in the red flour beetle. **(A,B)** Nucleotide components of 5′- and 3′-end cleavage sites of the top 1.0% of small RNAs for ds*EGFP*-720 in *Tribolium castaneum*. **(C,D)** Nucleotide components of 5′- and 3′-end cleavage sites of the top 1.0% of small RNAs for ds*Actin* in *T. castaneum*.

### The GGU Cleavage Site May Be a Universal Phenomenon in Lepidoptera

To explore whether this GGU cleavage site was universal, we selected an exogenous *EGFP* gene to synthesize dsRNA (ds*EGFP*-720). Then, for each gram of insect body weight for Asian corn borer or cotton bollworm larva, ds*EGFP*-720 was injected. The method of small RNA sequencing and re-mapping on the *EGFP* gene sequence was performed as described above (sequencing data are listed in Supplementary Tables 2–5). The small RNA mapping results indicated that the two different Lepidoptera insects have similar processing modes for this exogenous dsRNA sequence (Figure 3A). The nucleotide analysis of the small RNAs 5′- and 3′-ends also indicated that GGU is a hot point of dsRNA processing to siRNA in both lepidopteron insects (Figures 3B–E and Supplementary Figure 6).

### Major Difference Between the dsRNA-Processing Modes of Coleoptera and Lepidoptera

To further investigate the GGU digestion site in a different insect order, one fifth instar red flour beetle (*T. castaneum*) larva was injected with either ds*EGFP*-720 or ds*Actin* (GenBank accession no. XM_008201747) (0.04 mg dsRNA per gram insect body weight). The sample collection and small RNA sequence analysis were performed as described above (sequencing data are listed in Supplementary Tables 2–5).

To our surprise the GGU site was not found among the top 10 three-nucleotide combinations at 5′- and/or 3′-end cleavage sites in *Tribolium*. Instead, AAG, GUU, GAU, and GUG were the major three-nucleotide combinations at those positions (Figures 4A–D). Additionally, the site “5′-2” before the small RNA 5′-end contained mainly U and A (ds*EGFP*-720: 86.6%; ds*Actin*: 96.0%). Similarly, the site “-2” on the small RNA 3′-end also contained U and A (ds*EGFP*-720: 76.3%; ds*Actin*: 84.4%) (Figures 5A–D). This discovery means that enzyme restriction sites in siRNA processing in Coleoptera insects have a high conservation base of A/U. These results not only indicate the diversity of small RNA cleavage sites in the red flour beetle, but also imply that small RNAs are more easily processed at these kinds of nucleotide sites.

## Discussion

In this research, using high-throughput small RNA sequencing and bioinformatics analyses, we discovered that the same dsRNA sequence segments, no matter their lengths, undergo a similar *in vivo* siRNA-processing mode (Figures 1B, 3A). This result also implies that the dsRNA nucleotide sequence determines the siRNA type and amount. In addition, dsRNA produced siRNAs have strong base bias, GGU is the preferred recognition and cleavage sites when dsRNA is processed into siRNA in Lepidoptera (Figures 2, 3). However, the recognition and cleavage sites are more diverse in Coleoptera (Figures 4, 5). These results help to explain why the RNAi efficiency is so difference between these two insect orders (Terenius et al., 2011; Ivashuta et al., 2015; Joga et al., 2016).

Previous *in vitro* studies led to the 3′- and 5′-counting rules for dsRNA-processing models using the Dicer enzyme (Zhang et al., 2004; MacRae et al., 2007; Park et al., 2011). These rules, based on northern blot results, can explain siRNA lengths but are unable to distinguish nucleotide sequences (Gu et al., 2012). Moreover, previous studies usually selected relatively shorter dsRNAs for northern blot analysis, resulting in a limited spectrum of nucleotides sequences. To address these deficiencies, we used small RNA sequencing and bioinformatics analyses to extend the base composition range. We noticed that the small RNA peaks were not always produced at the 5′- or 3′-end, and this was mainly related to the nucleotide sequence components. Sequence cleavage preference had been shown in RNase III family proteins, different Dicer-like enzymes in *Paramecium* have different cleavage preference sites (Hoehener et al., 2018), and BsMiniIII in *B. subtilis* has a strong preference for ACCU/AGGU as a cleavage site (Glow et al., 2016). These results are consistent with our high-throughput sequencing analysis. Although our result is the processing mode of dsRNA *in vivo*, dsRNA is not exclusively performed by RNase III family members. It can also be accomplished by the cooperative actions of several enzymes, such as a specific exo- or endo-ribonuclease. In addition to being cleaved by an RNase III protein family member, dsRNA can also be degraded by some nucleases, such as RNase A and REase (Starega-Roslan et al., 2015; Guan et al., 2018). However, the processing of dsRNA into siRNAs has its own regularity in each species, even in the complicated *in vivo* environment. These result confirmed that the *in vivo* dsRNA processing has some regularities.

The regularity of the *in vivo* siRNA-processing mode will help in designing effective dsRNA segments for RNAi technology. Previous studies failed to discern gene segments when designing dsRNA. A study on *Acyrthosiphon pisum* showed that there was no significant difference when designing dsRNA based on the 5′ or 3′-end for the hunchback gene (Mao and Zeng, 2014). Experiments in *Aedes aegypti* showed that dsRNA was designed based on the 3′-end of the *apoptotic* gene, resulting in a higher mortality rate than those based on the 5′-end (Pridgeon et al., 2008). However, studies on *Litopenaeus vannamei* showed that dsRNA designed based on the 5′-end was more effective against antiviral effects (Loy et al., 2012). These results suggest that the RNAi effect of the 5′ or 3′-end segment as dsRNA templates vary among genes (Scott et al., 2013). According to our finding, for more effective RNAi, segments that easily produce small RNA peaks should be selected as dsRNA targets. GGU is a preferred cleavage site in Lepidoptera (Figures 2, 3). Most small RNA peaks have one or more GGU nucleotide residues (Supplementary Figure 5). Once the GGU were mutated, the small RNA peaks were lost (Figure 2G). However, in Coleoptera, the siRNA’s 5′ or 3′-end nucleotide residues are more diverse and GGU is not among the top 10 nucleotide combinations (Figures 4, 5). Most results indicated that members of Coleopteran are more sensitive to RNAi than those of Lepidoptera insects (Terenius et al., 2011; Ivashuta et al., 2015; Joga et al., 2016). Thus, the difference in RNAi efficiencies between these two insect orders may result from a difference in their genomes’ nucleotide compositions, the codon bias of their genes (Behura and Severson, 2012), enzyme-substrate contacts (Glow et al., 2016), RNAi pathway-related gene (Dowling et al., 2016), or various environmental differences that result in differences in dsRNA stability (Spit et al., 2017). Here, we discovered that there was a large difference in the dsRNA’s cleavage between Lepidoptera and Coleoptera insects. The dsRNA-processing sites are more diverse in Coleoptera and this insect order is sensitive to RNAi; our discovery supplies new evidence for RNAi efficiency. Discerning these regularities will increase the understanding RNAi mechanisms and aid in the design of effective dsRNAs for *in vitro* studies and applications.

## Author Contributions

XM and RG designed research. RG and SH performed research. RG and HL analyzed the data. XM and ZS wrote and revised the paper.

## Conflict of Interest Statement

The authors declare that the research was conducted in the absence of any commercial or financial relationships that could be construed as a potential conflict of interest.
