# Supplementary material for: The in vivo dsRNA Cleavage Has Sequence Preference in Insects
Source: Front Physiol. 2018 Dec 10;9:1768. doi: 10.3389/fphys.2018.01768 (PMC6295558; doi:10.3389/fphys.2018.01768)
Supplement: Supplementary file 6 [file Table_6.pdf]

Supplementary Material

The *in vivo* dsRNA cleavage has sequence preference in insects

Ruobing Guan[1, 2#], Shaoru Hu[1,3#], Haichao Li[1], Zhenying Shi[1] and Xuexia Miao[1*]

[1] Key Laboratory of Insect Developmental and Evolutionary Biology, Institute of Plant Physiology and Ecology, Shanghai Institutes for Biological Sciences, Chinese Academy of Sciences, Shanghai 200032, China, [2] State key Laboratory of Wheat and Maize Crop Science, College of Plant Protection, Henan Agricultural University, Zhengzhou, China, [3] University of the Chinese Academy of Sciences, Beijing 100049, China

***Corresponding author:** E-mail: xxm@sibs.ac.cn

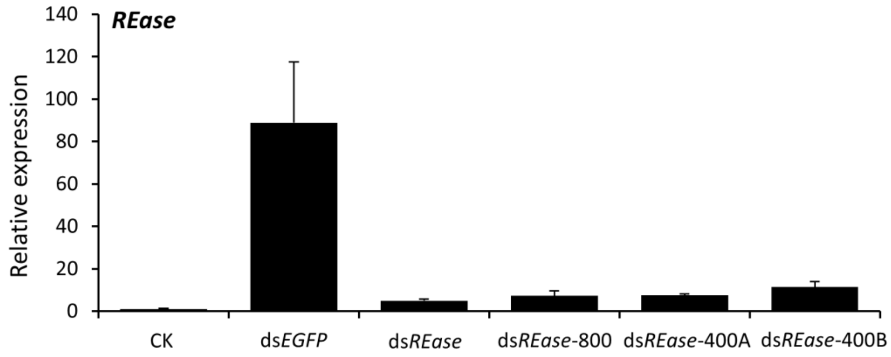

Supplementary Figure 1. The *REase* gene expression level in ds*EGFP* and ds*REase* treatments.

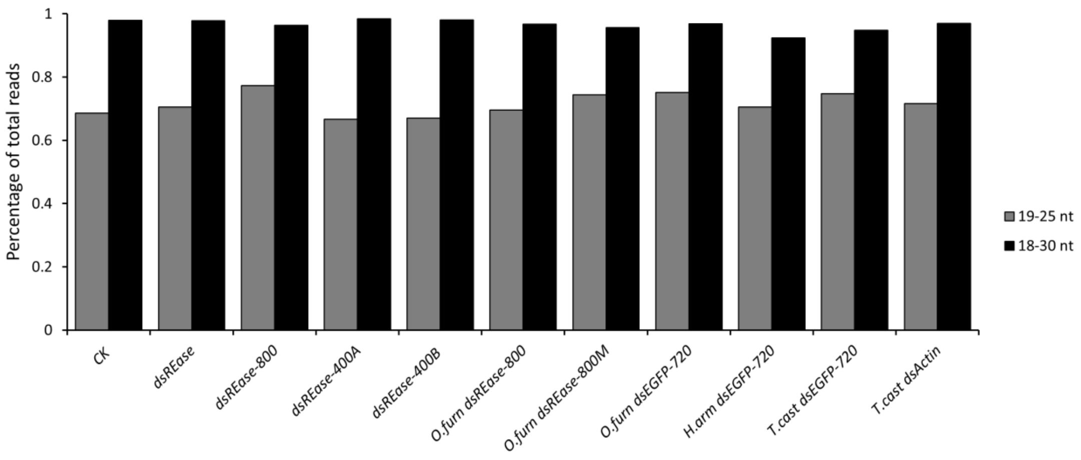

Supplementary Figure 2. Distribution of 19–25 nt and 18–30 nt small RNAs under

different treatment conditions.

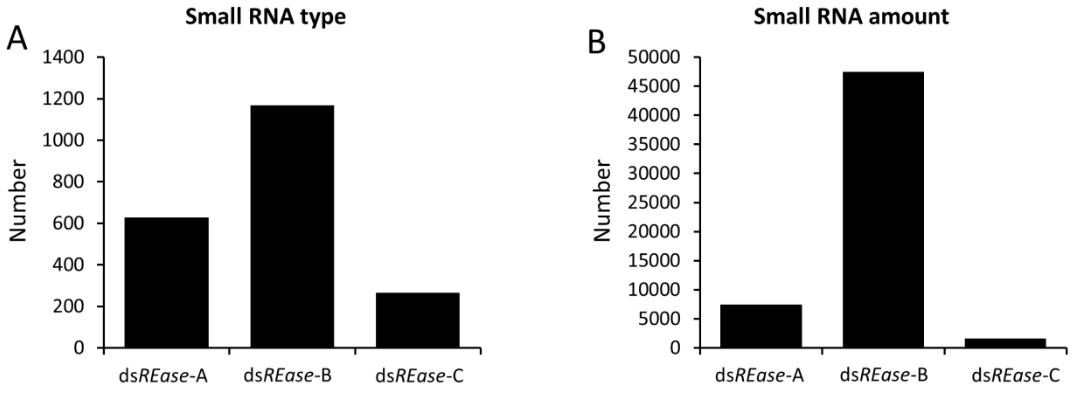

Supplementary Figure 3. Statistics regarding small RNA type and amount at different sequence positions.

(A) Small RNA type in three different sequence position of ds*REase*-A, ds*REase*-B, and ds*REase*-C showed in Figure 1B. (B) Small RNA amount in three different sequence position of ds*REase*-A, ds*REase*-B, and ds*REase*-C showed in Figure 1B.

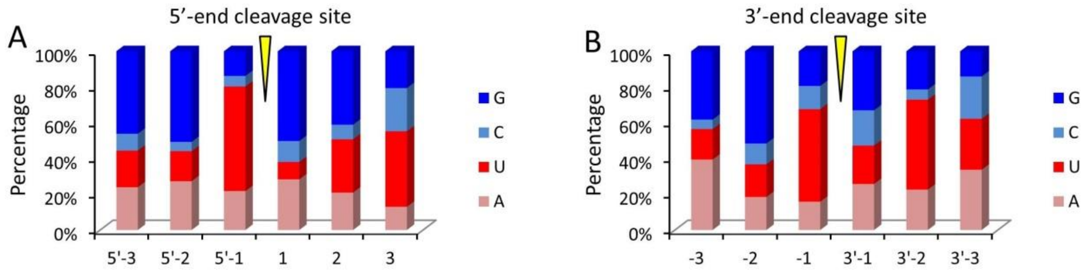

Supplementary Figure 4. Nucleic acid compositions of 5′- and 3’-ends cleavage sites in the top 1.0% of small RNAs after ds*REase* treatment.

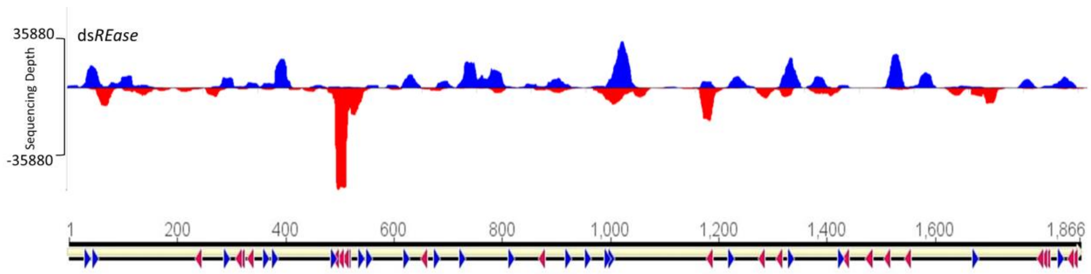

Supplementary Figure 5. Relationship between GGU sites in the *REase* sequence and the *in vivo* processing model of ds*REase*. The graph of upper panel: x-axis represents the *REase* sequence, and the y-axis represents the depth of sequencing (amount of mapped small RNA).The sense chain is marked in blue, and the antisense chain is marked in red. The graph of lower panel: red arrow head represents GGU sites on the sense chain, red arrow head represents the GGU sites on the antisense chain.

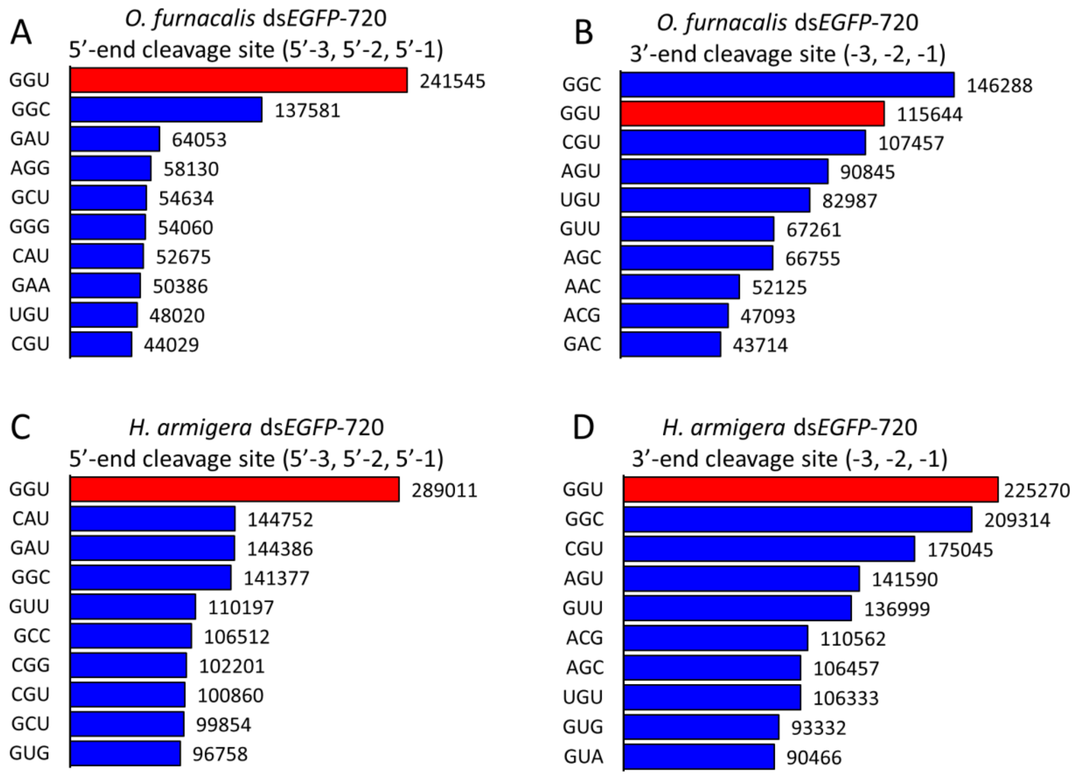

Supplementary Figure 6. Numbers of different nucleotide compositions of 5′- and 3′-ends cleavage sites in the top 1.0% of small RNAs of *Ostrinia furnacalis* ds*EGFP*-720 and *Helicoverpa armigera* ds*EGFP*-720.
